# The Effect and Safety of 5-HT_1F_ Receptor Agonist Lasmiditan on Migraine: A Systematic Review and Meta-Analysis

**DOI:** 10.1155/2021/6663591

**Published:** 2021-10-07

**Authors:** Ping Gu, Cheng Chen, Qian Wu, Changhong Dong, Teng Wang, Qi Wan, Xin Dong

**Affiliations:** ^1^Department of Neurology, The First Affiliated Hospital of Nanjing Medical University, 300 Guangzhou Road, Nanjing, Jiangsu Province, China; ^2^Department of Neurology, The Second Affiliated Hospital of Wannan Medical College, 10 Kangfu Road, Wuhu, Anhui Province, China; ^3^Department of Clinical Medicine, Xuzhou Medical University, 209 Tongshan Road, Xuzhou, Jiangsu Province, China

## Abstract

**Background:**

Migraine has a great impact on public health. Current acute therapies do not satisfy all migraineurs. The novel serotonin 5-HT_1F_ receptor agonist appears more promising for aborting migraine attacks.

**Objective:**

To evaluate the clinical efficacy and safety of lasmiditan in treating acute migraine attacks.

**Methods:**

The literature search was performed in PubMed, Embase, and the Cochrane Central Register of Controlled Trials for randomized controlled trials (RCTs) which assessed the effect and safety of lasmiditan on migraine. The risk of bias was assessed using the Cochrane Collaboration's risk of bias tool. Results were extracted and pooled as risk ratios (RRs) with a fixed or random-effects model.

**Results:**

Based on the four included RCTs, pooled estimates showed that lasmiditan with the 50 mg, 100 mg, and 200 mg doses was superior to placebo at 2 h after the first dose in terms of pain freedom, absence of migraine-associated symptoms, headache relief, no/mild disability, and global impression of change (very much/much better) (RRs ranged from 1.13 to 1.96), except for nausea-free and vomiting-free. Both lasmiditan 100 mg and 200 mg resulted in significantly fewer patients using rescue medication (100 mg: RR = 0.75, 95% CI (0.61, 0.92), *P* = 0.007; 200 mg: RR = 0.81, 95% CI (0.66, 0.99), *P* = 0.04) at 2-24 h postdose, compared with placebo. Safety data showed that the proportion of patients reporting at least one treatment-emergent adverse event (TEAE) and the incidence of most common TEAEs such as dizziness, paresthesia, fatigue, somnolence, and nausea was higher in the lasmiditan groups (50 mg, 100 mg, and 200 mg), compared with placebo. There was no significant difference between lasmiditan and placebo in terms of cardiovascular-related TEAEs (RR = 2.75, 95% CI (0.81, 9.37), *P* = 0.11). Compared with lasmiditan 100 mg, lasmiditan 200 mg was more effective in pain freedom at 2 h after the first dose (RR = 0.83, 95% CI (0.74, 0.94), *P* = 0.004) but associated with a higher risk of reporting at least one TEAE (RR = 0.88, 95% CI (0.81, 0.96), *P* = 0.006).

**Conclusions:**

Lasmiditan with the 50 mg, 100 mg, and 200 mg doses are effective and safe in acute migraine treatment. Lasmiditan 200 mg is more effective than lasmiditan 100 mg in pain freedom, while lasmiditan 100 mg is better tolerated in the short-term follow-up. Further larger sample-size RCTs are required to verify the applicability and tolerability in the long term.

## 1. Introduction

Migraine is a common chronic disabling neurological disorder, characterized by unilateral moderate to severe headache attacks and accompanied by nausea, vomiting, photophobia, and phonophobia [1]. In the Global Burden of Disease Study 2016, migraine is reported as the second most disabling disorder, which reduced quality of life and increased the use of health resources [2, 3]. Acute migraine treatment is aimed at relieving pain and abort headache attack [4]. The present acute migraine therapy contains specific (ergots, triptans) and nonspecific drugs (nonsteroidal anti-inflammatory drugs) [5]. However, it should be noted that 40% of episodic migraineurs are not satisfied with the effects of acute treatments, which might lead to a high risk of medication overuse headache and exacerbations of headache [6].

Triptans acting on the 5-HT_1B/1D_ receptors are the first-line choice in the acute treatment for migraine attack currently, which were introduced into the clinical application in the early 1990s and represented a milestone in acute antimigraine therapy [7]. All oral triptans at marketed doses were effective by constricting extracerebral and intracranial vessels, inhibiting trigeminal neurons, and blocking plasma protein extravasation [8]. However, up to 35% of patients with episodic migraine do not benefit from triptans. Also, triptans are contraindicated in migraineurs with myocardial infarction, coronary artery disease, stroke, uncontrolled hypertension, and vasculitis because of their potential for vasoconstriction [9].

Although the pathophysiology of migraine has not been fully understood, trigeminal pathways have been proposed to be involved in the pathogenesis of migraine for decades [10]. Neural inhibition of the 5-HT_1F_ receptor expressed in the trigeminal ganglia would provide a potential alternative to treat acute migraine with nonvascular mechanism [11]. Lasmiditan (COL-144, LY573144), a novel highly selective 5-HT_1F_ receptor agonist, is currently in phase III trials for acute migraine therapy. It has a much higher affinity (>470-fold) for the 5-HT_1F_ than 5-HT_1B/D_ receptors in vivo binding studies [12]. Phase II and phase III clinical trials of lasmiditan showed a significantly better treatment response and a higher incidence of the central nervous system- (CNS-) related adverse events than the placebo, especially in high-dose groups [13]. The US Food and Drug Administration has approved the use of 100 mg and 200 mg doses of lasmiditan for acute treatment of migraine attacks with or without aura [14]. However, there is no systematic evaluation of the clinical efficacy and safety of different dosages of lasmiditan quantificationally. Thus, we performed a systematic review and meta-analysis of randomized controlled trials (RCTs) on the efficacy and safety of different doses of lasmiditan for acute migraine treatment.

## 2. Methods

### 2.1. Study Design and Registration

The meta-analysis adhered to the requirements of the Preferred Reporting Items for Systematic Reviews and Meta-analysis (PRISMA) statement (S1 File). This review protocol was registered in the International Prospective System Reviews (No. CRD42020188661).

### 2.2. Search Strategy and Inclusion Criteria

Two reviewers (Dong and Chen) independently searched PubMed, Embase, and the Cochrane Central Register of Controlled Trials to identify the studies published up to May 2020. The complete search strategy was presented in S2 File. We further tracked the reference lists of retrieved articles and identified relevant grey literature and conference abstracts. We screened all trials satisfying the following inclusion criteria: treatment of migraine attacks in adult migraineurs with or without aura defined according to the International Classification of Headache Disorders, third edition or second edition; treatment with an oral or intravenous lasmiditan; randomized, double-blinded, controlled (placebo) clinical trials; aimed to evaluate the efficacy and safety of lasmiditan for acute treatment of migraine. The disagreement between the two reviewers would be resolved by consultation with a third author (Gu).

### 2.3. Quality Assessment

Two investigators assessed the methodological quality of included studies, respectively, by using the Cochrane Collaboration's risk of bias tool for systematic reviews [15, 16]. The domains in the Cochrane Collaboration's tool for assessing the risk of bias included random sequence generation, allocation concealment, blinding of participants and personnel, blinding of outcome assessment, incomplete outcome data, selective reporting, and other biases. The bias of each item was judged as a low risk of bias, a high risk of bias, or an unclear risk of bias. Disagreements were resolved by discussing with the corresponding author (Wan).

### 2.4. Data Extractions

Efficacy data and adverse events for lasmiditan and placebo were extracted independently by two reviewers (Dong and Chen) using the predefined data extraction form. Only data above the dose (≥20 mg intravenous or 50 mg oral) in the experimental group was included in this meta-analysis. Two different delivery routes, including oral and intravenous, were adopted in our included trials. Conversion of intravenous doses into oral doses was achieved according to bioavailability, reported as approximately 40% [17]. Any discrepancies among the two reviewers were resolved through discussion or consensus with a third reviewer (Gu). We extracted the characteristics of each RCT recorded as follows: first author, publication year, diagnostic criteria, study population characteristics (age, sex, and ethnicity), intervention (placebo or lasmiditan with different doses), and baseline headache characteristics of migraineurs (migraine attacks per month). The primary outcome included pain freedom and absence of migraine-associated symptoms at 2 h after the first dose. The secondary outcomes included headache relief, no/mild disability, the global impression of change (very much/much better) at 2 h after the first dose, and rescue medication from 2 to 24 h postdose. Treatment-emergent adverse events (TEAEs) after the first dose were recorded to assess the safety of lasmiditan, including the proportion of patients with TEAEs (at least one), the incidence of the most common TEAEs (dizziness, paresthesia, fatigue, lethargy, and nausea), and cardiovascular-related TEAEs. The related data reported incompletely in the articles was extracted from the Clinical trials.gov.

### 2.5. Statistical Analysis

The data about the responder rate in efficacy and adverse events were dichotomous. The relative risk (RR) with 95% confidence intervals (CIs) was used to estimate the efficacy and safety using the Mantel-Haenszel (M-H) method. The statistical heterogeneity was assessed by the chi-squared test, and its extent was measured by the *I*^2^ statistic. For the chi-squared test, a significance level was set at *P* < 0.10. If the *I*^2^ value was greater than 50%, we believed that there might be a significant heterogeneity, and *I*^2^ less than 50% indicated no significant heterogeneity [18]. When there was no significant heterogeneity, we used a fixed-effects model for pooling the data; otherwise, a random-effects model was used. Sensitivity analysis attempted to identify the source of heterogeneity by excluding any single hazard ratio from the analysis data subsequently. According to the frequently investigated doses of lasmiditan (50 mg, 100 mg, and 200 mg), we performed a pooled analysis to assess the effects of lasmiditan against placebo and between different dosages of lasmiditan. Metaregression would not be considered in this meta-analysis because of fewer included studies (less than 10). We did not access publication bias by a visual assessment of a funnel plot, Egger, or Begg test attributing to fewer included trials (less than 10) [19, 20]. All meta-analyses were performed using the software RevMan 5.3 (Cochrane Collaboration, Oxford, England). *P* < 0.05 was considered statistically significant.

## 3. Results

### 3.1. Study Selection and Inclusion

**Figure 1:**
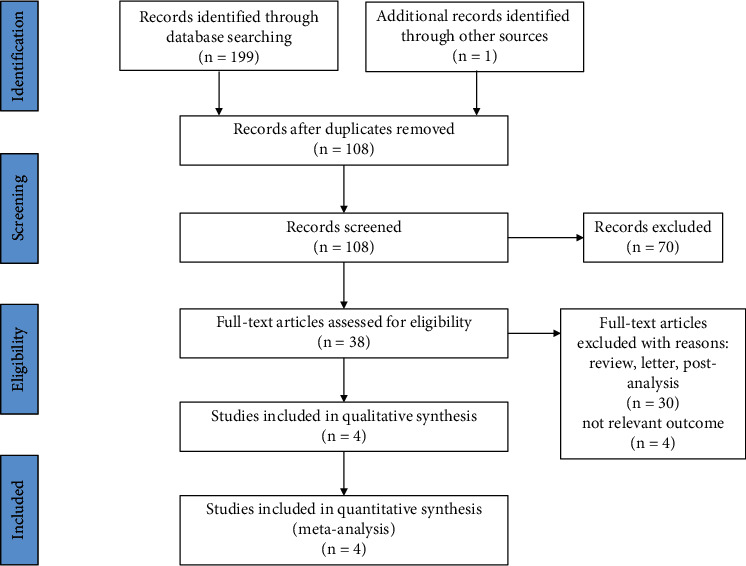
Process of identifying eligible studies for the meta-analysis.

The trial selection process was presented in the PRISMA flow chart (Figure 1). We identified 200 articles in the database searches according to the search strategy. 108 articles remained after removing the duplicated publication, and only 38 articles remained after title and abstract screening. 34 articles were excluded because of their nature as review, letter, or postanalysis of clinical trials. Ultimately, four RCTs involving 4920 participants were included in this meta-analysis [21–24].

### 3.2. Study Characteristics

All four trials were multicenter, 1double-blind RCTs, published between 2010 and 2019 [21–24]. Participants were diagnosed with migraine based on the IHS criteria. All eligible patients were adults (≥18 years), with a mean age of 42.17 years, and 84.29% were female. The mean migraine attacks per month reported at baseline was 5.06. Lasmiditan was used to treat a single acute migraine attack in all trials. A total of 764 patients were randomized to lasmiditan 50 mg (oral and intravenous administration), 1347 to lasmiditan 100 mg, 1329 to lasmiditan 200 mg, and 1390 to placebo. Three studies adopted oral delivery routes, and only one trial evaluated the efficacy of intravenous lasmiditan. The characteristics of the four included studies are summarized in Table 1.

### 3.3. Quality of the Included Studies

**Figure 2:**
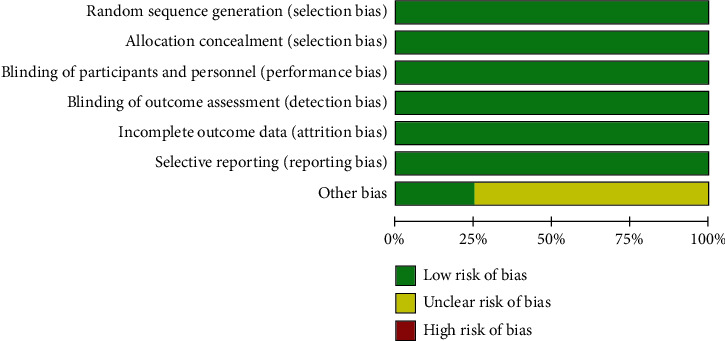
Risk of bias for included trials.

The quality assessments of the included four RCTs are summarized in Figure 2, according to the Cochrane Collaboration's tool for assessing the risk of bias. As shown in Figure 2, all subjects were randomized to receive lasmiditan and placebo according to the randomized sequence generated by central randomization or computer randomization system. All studies reported allocation concealment. Therefore, we judged these studies as having a low risk of selection bias. Each subcenter investigator and patient were masked from treatment selection during the study. Only one study by Ferrari et al. [21] reported that the pharmacist was aware of the drug dilution, but the pharmacist was independent of the investigator. Thus, the risk of performance bias was judged as low. We rated all studies at low risk of detection bias since all studies used the headache diary to record responses before and after the study drug intake. The loss rate was balanced among groups and did not exceed 10%. The intention-to-treat analyses were utilized in all studies. Therefore, the risk of attrition bias and reporting bias were judged as low in all included studies. Due to the dose-adaptation design in the study by Ferrari et al. [21] and many patients with vascular risks in the studies by Kuca et al. [23] and Goadsby et al. [24], other biases in these included studies were considered to be at unclear risk. Overall, the included studies were suitable for the meta-analysis of the effect and safety of lasmiditan for migraine.

### 3.4. Primary Outcomes

#### 3.4.1. Pain Freedom at 2 h

**Figure 3:**
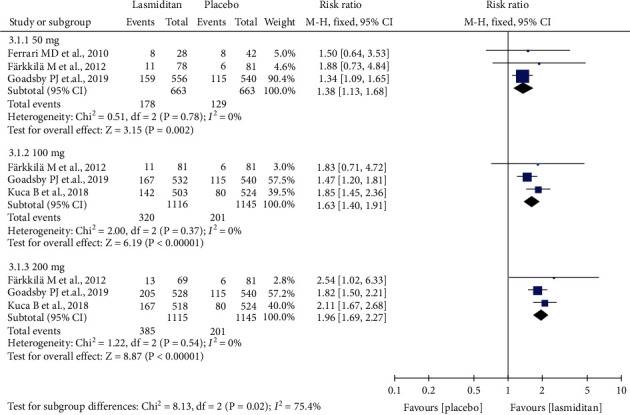
Forest plot: responder rate for pain freedom at 2 h of lasmiditan versus placebo after the first dose (50 mg, 100 mg, and 200 mg).

**Figure 4:**
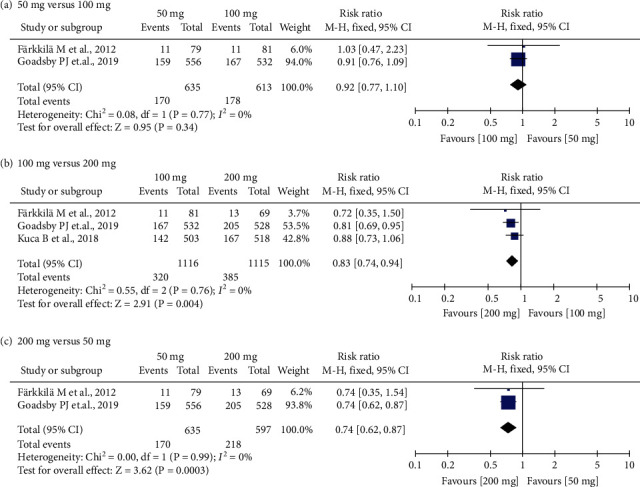
Forest plot: responder rate for pain freedom between different dosage comparisons at 2 h after the first dose.

Pain freedom at 2 h was reported in all included trials. Pain freedom in migraineurs at 2 h referred to headache pain-free at 2 h after the first dose and before any rescue medication [25]. The pooled data analyzed with a fixed-effects model demonstrated that the percentage of patients achieving pain-free was higher in the 50 mg, 100 mg, and 200 mg lasmiditan dose groups than in the placebo group (50 mg: RR = 1.38, 95% CI (1.13, 1.68), *P* = 0.002; 100 mg: RR = 1.63, 95% CI (1.40, 1.91), *P* < 0.00001; 200 mg: RR = 1.96, 95% CI (1.69, 2.27), *P* < 0.00001) (Figure 3). Both the 50 mg (RR = 0.74, 95% CI (0.62, 0.87), *P* = 0.0003) and 100 mg (RR = 0.83, 95% CI (0.74, 0.94), *P* = 0.004) doses of lasmiditan were inferior to 200 mg dose of lasmiditan in terms of pain freedom at 2 h after the first dose (Figure 4). No significant difference was detected between the 50 mg and 100 mg lasmiditan dose group, based on two RCTs. There was no heterogeneity between studies (*I*^2^ = 0%).

#### 3.4.2. Absence of Migraine-Associated Symptoms at 2 h

Migraine-associated symptoms included nausea, vomiting, photophobia, and photophobia were reported in four included RCTs. Compared with placebo, migraineurs treated with lasmiditan reported a higher response rate of phonophobia-free (50 mg: RR = 1.13, 95% CI (1.05, 1.22), *P* = 0.002; 100 mg: *RR* = 1.16, 95% CI (1.10, 1.22), *P* < 0.00001; 200 mg: RR = 1.16, 95% CI (1.10, 1.22), *P* < 0.00001) and photophobia-free (50 mg: RR = 1.19, 95% CI (1.04, 1.37), *P* = 0.01; 100 mg: RR = 1.35, 95% CI (1.17, 1.57), *P* < 0.0001; 200 mg: RR = 1.30, 95% CI (1.04, 1.40), *P* < 0.00001) at 2 hours after dosing (Table 2). However, the RRs for nausea-free and vomiting-free did not favor lasmiditan over placebo. Both lasmiditan 100 mg (RR = 0.91, 95% CI (0.84, 0.98), *P* = 0.02) and 200 mg (RR = 1.12, 95% CI (1.04, 1.22), *P* = 0.005) resulted in a significantly higher rate of photophobia-free at 2 h after the first dose, as compared with lasmiditan 50 mg. There was significant heterogeneity in the estimates of 100 mg lasmiditan for photophobia-free (*I*^2^ = 72%). Sensitivity analysis showed that the heterogeneity could be resolved by excluding the study by Färkkilä et al. [22] from the pooled data with little change of the overall effect. The heterogeneity might be caused by the characteristics of participants, relatively small sample size, and the discrepancy in the evaluation criteria in the trial by Färkkilä et al. [22]. The headache severity and the proportion of migraine without aura were higher than in the other included trials.

### 3.5. Secondary Outcomes

#### 3.5.1. Headache Relief at 2 h

The RRs of headache relief at 2 h after treatment favored lasmiditan over placebo (50 mg: RR = 1.27, 95% CI (1.15, 1.41), *P* < 0.00001; 100 mg: RR = 1.51, 95% CI (1.25, 1.82), *P* < 0.0001; 200 mg: RR = 1.41, 95% CI (1.28, 1.54), *P* < 0.00001) (Table 3). There was statistically significant heterogeneity in headache relief among trials when compared lasmiditan 100 mg with placebo (*I*^2^ = 75%). Sensitivity analysis showed that heterogeneity could be resolved by removing the study by Färkkilä et al. [22] from the pooled data, with no significant effect on the pooled estimate. No significant difference was detected among any dose comparisons in headache relief at 2 h (Table 3).

#### 3.5.2. No/Mild Disability at 2 h

As shown in Table 3, all three doses of lasmiditan (50 mg, 100 mg, and 200 mg) resulted in significantly more patients achieving no/mild disability at 2 h after the first dose (50 mg: RR = 1.13, 95% CI (1.03, 1.25), *P* = 0.01; 100 mg:RR = 1.23, 95% CI (1.08, 1.40), *P* = 0.001; 200 mg: RR = 1.16, 95% CI (1.03, 1.30), *P* = 0.01), compared to the placebo group. Compared with the 50 mg lasmiditan group, the proportion of patients who achieved no/mild disability at 2 h (RR = 0.90, 95% CI (0.82, 0.98), *P* = 0.01) was significantly higher in the 100 mg lasmiditan group. No significant differences were detected among other dose comparisons. We found significant heterogeneity among the studies for comparing lasmiditan 100 mg with placebo. Sensitivity analysis showed that the heterogeneity could be reduced by excluding the study by Färkkilä et al. [22] from the pooled data with little change of the overall outcome.

#### 3.5.3. Global Impression of Change (Very Much/Much Better) at 2 h

Lasmiditan 50 mg (RR = 1.33, 95% CI (1.13, 1.55), *P* = 0.0004), 100 mg (RR = 1.60, 95% CI (1.42, 1.81), *P* < 0.00001), and 200 mg (RR = 1.62, 95% CI (1.43, 1.82), *P* < 0.00001) were associated with more patients achieving global impression of change (very much/much better), compared with placebo at 2 h after the first dose (Table 3). There was no significant heterogeneity between studies. Lasmiditan 100 mg (RR = 0.86, 95% CI (0.75, 0.99), *P* = 0.04) and 200 mg (RR = 1.16, 95% CI (1.01, 1.34), *P* = 0.03) resulted in more patients achieving global impression of change (very much/much better) at 2 h after the first dose when compared with lasmiditan 50 mg. No significant difference was detected when comparing lasmiditan 100 mg with 200 mg, based on three RCTs (Table 3).

#### 3.5.4. Use of Rescue Medication (2–24 h)

Compared with placebo, lasmiditan 100 mg (RR = 0.75, 95% CI (0.61, 0.92), *P* = 0.007) and 200 mg (RR = 0.81, 95% CI (0.66, 0.99), *P* = 0.04) resulted in significantly fewer patients using rescue medication at 2-24 h. There was no heterogeneity between studies (*I*^2^ = 0%). No significant difference was detected between lasmiditan 50 mg and placebo, or among other dose comparisons in the proportion of patients using rescue medication (Table 3).

### 3.6. Adverse Events

No deaths were reported in the four trials. All the included RCTs reported the incidence rates of the most common TEAEs. In this meta-analysis, three RCTs were evaluated for the proportion of patients who reported at least one TEAE, and two RCTs reported the cardiovascular-related TEAEs. The most common TEAEs in the trials after dosing were CNS-related TEAEs including dizziness, paresthesia, fatigue, somnolence, and nausea.

#### 3.6.1. At Least One TEAE

**Figure 5:**
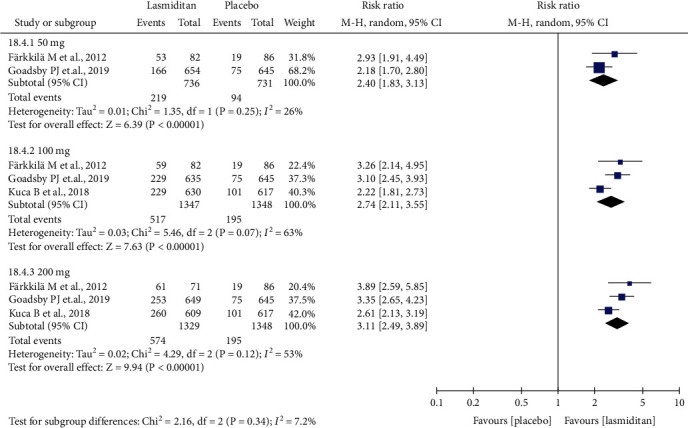
Forest plot: the proportion of migraineurs reporting TEAE (at least one) in the lasmiditan group (50 mg, 100 mg, and 200 mg) versus placebo after the first dose.

A higher proportion of patients reported at least one TEAE after the first dose in the 50 mg (RR = 2.40, 95% CI (1.83, 3.13), *P* < 0.00001), 100 mg (RR = 2.74, 95% CI (2.11, 3.55), *P* < 0.00001), and 200 mg lasmiditan dose groups (RR = 3.11, 95% CI (2.49, 3.89), *P* < 0.00001), as compared with the placebo group (Figure 5). Compared with lasmiditan 200 mg, we found lasmiditan 50 mg (RR = 0.67, 95% CI (0.59, 0.77), *P* < 0.00001) and 100 mg (RR = 0.88, 95% CI (0.81, 0.96), *P* = 0.006) resulted in significantly fewer patients reporting at least one TEAE (S3 File). There was significant between-study heterogeneity (100 mg: *I*^2^ = 63%; 200 mg: *I*^2^ = 53%). Removing the study by Kuca et al. [23] from the pooled data, the heterogeneity could be reduced.

#### 3.6.2. Most Common TEAEs

As summarized in Table 4, patients treated with lasmiditan at each dose reported higher incidence rates of the most common TEAEs (dizziness, paresthesia, fatigue, somnolence, and nausea) than with placebo, except for nausea in the lasmiditan 100 mg dose group. A higher proportion of patients reported dizziness, paresthesia, and fatigue after the first dose in the 200 mg lasmiditan dose group compared to the 50 mg lasmiditan dose group. Compared with the lasmiditan 50 mg group, patients in the lasmiditan 100 mg group had a higher incidence rate of paresthesia. We found no significant difference in the incidence rates of the most common TEAEs when comparing lasmiditan 100 mg with 200 mg. There was significant heterogeneity between the trials in the estimates of 100 mg lasmiditan for dizziness and nausea after dosing. Removing the study by Kuca et al. [23], the heterogeneity between the studies could be reduced.

#### 3.6.3. Cardiovascular-Related TEAEs

**Figure 6:**
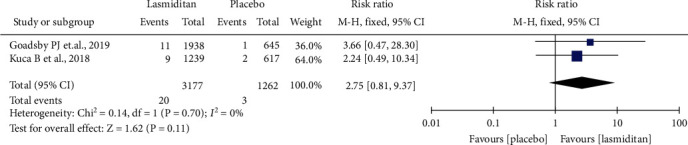
Forest plot: the incidence of cardiovascular-related TEAEs after the first dose.

Two RCTs reported the cardiovascular-related TEAEs. The risk of cardiovascular-related TEAEs was not significantly higher in lasmiditan treatment groups compared with placebo (RR = 2.75, 95% CI (0.81, 9.37), *P* = 0.11). There was no heterogeneity between studies (*I*^2^ = 0%) (Figure 6).

## 4. Discussion

Our meta-analysis presented that lasmiditan is a promising therapeutic option for aborting migraine attacks by assessing the efficacy and safety of lasmiditan. Lasmiditan, an emerging selective 5-HT_1F_ receptor agonist, could cross the blood-brain barrier (BBB) and inhibit dural plasma protein extravasation by acting on the 5-HT_1F_ receptors expressed in trigeminal neurons, trigeminal ganglion, and trigeminal caudal nucleus, which are not expressed in vascular endothelial cells or smooth muscle cells of the brain [26]. The clinical efficacy of lasmiditan without vasoconstrictive activity implies that vasoconstriction is not essential for antimigraine therapy and strengthens the established neurogenic hypothesis that the trigeminal nerve system may be involved in central neuronal mechanisms of migraine pathophysiology. Considering the structural and pharmacological differences between lasmiditan and triptans [12, 27], lasmiditan is probably more suitable for migraineurs with insufficient response or contraindications for triptan.

In the case of outcomes being reported at multiple time points in some trials, the outcome measures of efficacy at 2 h after drug intake would be extracted because of the maximum serum concentration following oral administration [17]. Lasmiditan with the 50 mg, 100 mg, and 200 mg was superior to placebo in terms of pain freedom, photophobia-free, phonophobia-free, headache relief, no/mild disability, and global impression of change in acute treatment of migraine at 2 h postdose except nausea-free and vomiting-free. Lasmiditan 100 mg or 200 mg had superior efficacy as measured by the use of rescue medication. Nausea and/or vomiting symptoms were not only migraine-associated symptoms but also drug-induced adverse effects, such as treatment-emergent nausea or vomiting postdose [25]. The difficulty in identifying whether nausea and/or vomiting resulted from unrelieved migraine-associated symptoms or unavoidable drug-induced adverse effects probably contributed to no advantage of lasmiditan for nausea-free and vomiting-free compared with placebo. Triptans produce a headache response at 2 h in 60% of migraine patients, and 30% of migraineurs are pain freedom at 2 h [8]. According to the overall pooled data, lasmiditan produces headache relief at 2 h in 61.05% of migraineurs, and 30.48% of migraineurs are pain freedom at 2 h. For lasmiditan, the effect of acute migraine treatment in terms of pain freedom and headache relief may be equivalent to triptans. Future well-designed studies should be provided for a head-to-head comparison between lasmiditan and triptans. This meta-analysis evaluated the short-term outcomes for a single attack of migraine with lasmiditan. Studies with continued dosing of lasmiditan in the long-term follow-up should be conducted in the future. Currently, results in an open-label study have addressed that lasmiditan was safe and efficacious for the acute treatment of migraine from the long-term data [28].

In four RCTs, lasmiditan did not show triptan-like side effects including jaw, neck, and chest symptoms. Safety parameters such as heart rate, blood pressure, 12-lead electrocardiogram (ECG), hematology, biochemistry, and urine analysis did not show any clinically drug-related pathological abnormalities across the drug groups. No deaths related to lasmiditan were reported in the trials. In general, the safety profile of lasmiditan was satisfactory. We found that significantly more patients dosed with lasmiditan. 50 mg, 100 mg, and 200 mg reported at least one TEAEs than patients receiving placebo. Most common TEAEs were CNS-related owing to central nervous system penetration. Most CNS-related TEAEs were mild to moderate in severity and short in duration, while varied CNS-related TEAEs had a high rate of occurrence (e.g., dizziness, paresthesia, fatigue, somnolence, and nausea). Dizziness symptom followed by vertigo and fatigue was the main complaint in CNS-related TEAEs. Lasmiditan crosses the BBB and binds to 5-HT_1F_ receptors located in trigeminal nerve terminals and other central or peripheral areas, but the specific site of action has not been definitely elucidated [12]. The study by Lucaites et al. [29] reported radioactive ligands, a potential tool to explore the localization and functions of the 5-HT_1F_ receptor, could bind significantly to 5-HT_1F_ receptors in the cerebellum, which could contribute to unwanted vertigo and dizziness symptom after drug administration. The incidence of CNS-related TEAEs is as high as 15% in some triptans [30], which seems to be more frequent in lasmiditan. Frequent occurrence of CNS-related TEAEs with triptans may reduce patient compliance, limit treatment initiation, and even impair workplace productivity and effectiveness despite the efficacy in aborting migraine attacks [30]. In a recent survey by the National Headache Foundation, up to 67% of patients reported delaying or avoiding their prescription medication for migraine due to concerns about side effects [31]. However, whether the high rate of CNS-related TEAEs may limit the clinical acceptability of lasmiditan still needs to be evaluated in long-term treatment studies. The pooled results of TEAEs should be interpreted with caution owing to the relatively high heterogeneity in reporting TEAEs, particularly for dizziness. The study by Färkkilä et al. [22] reported the differences between countries in the rates of dizziness, which suggested cultural and linguistic factors probably attributed to the heterogeneity of multicenter studies from different countries. In future studies, the procedure of reporting TEAEs should be modified to avoid possible overreporting of TEAEs.

The incidence of cardiovascular-related TEAEs reported by two studies was low. The cardiovascular events were generally mild or moderate in severity, and few were serious. Post hoc analysis for the incidence of cardiovascular events showed that patients with at least one cardiovascular risk factor (CVRF) enrolled by the two studies accounted for 78.8% at baseline. The pooled estimate showed no difference in the incidence of cardiovascular-related TEAEs between lasmiditan and placebo, which provided insight into the safety of lasmiditan in patients with CVRFs. The difference in the incidence of cardiovascular-related TEAEs between patients with CVRFs and without CVRFs needs to be further investigated in the future, although the post hoc analysis of these two studies in phase III showed no difference [32].

Our meta-analysis evaluated the efficacy and safety of lasmiditan in different dosages (50 mg, 100 mg, and 200 mg). We found a dose-related response of lasmiditan for pain freedom. Based on the results of this meta-analysis, we found that lasmiditan 100 mg and 200 mg are more effective than lasmiditan 50 mg in terms of pain-free, photophobia-free, and global impression of change (very much/much better), while lasmiditan 100 mg and 200 mg have no advantage over lasmiditan 50 mg in terms of headache relief, phonophobia-free, and use of rescue medication. Compared with lasmiditan 100 mg, lasmiditan 200 mg is more effective in achieving pain-free with a higher risk of reporting at least one TEAE. There are no significant differences between lasmiditan 100 mg and 200 mg in terms of headache relief, photophobia-free, phonophobia-free, no/mild disability, and global impression of change (very much/much better), use of rescue medication, and most common TEAEs. Consequently, lasmiditan with 200 mg dose provides superior efficacy to 100 mg in pain freedom at 2 h after the first dose. However, the clinical effect of the lasmiditan 200 mg should be balanced against the higher risk of reporting at least one TEAE.

Some limitations in this meta-analysis should be considered. Firstly, few eligible studies were included in this meta-analysis. Subgroup analysis about confounding factors, such as sex, ethnicity, migraine severity, and concomitant preventive medications, has not been conducted in included trials due to the insufficient available trials and data. Secondly, the long-term efficacy and safety of lasmiditan should be validated by following up the participants using drugs repeatedly in further studies. Third, various headache characteristics of participants and efficacy assessment standards across studies resulted in significant heterogeneity between the studies in the meta-analyses. Headache pain relief was defined as a reduction of moderate or severe pain to mild or no pain in the study by Färkkilä et al. [22], or a reduction in headache severity from mild to none in studies by Kuca et al. and Goadsby et al. [23, 24]. Finally, there was a lack of active control groups. Future RCTs are recommended to provide a head-to-head comparison between lasmiditan and other acute drugs such as triptans.

## 5. Conclusions

Lasmiditan is effective and safe for acute migraine treatment, despite the high incidence of TEAEs. The clinical effect of lasmiditan 200 mg is superior to lasmiditan 100 mg, while lasmiditan 100 mg is better tolerated in the short-term follow-up. Superiority clinical trials are necessary to verify the clinical superiority of lasmiditan over the traditional antimigraine drugs and triptans. Additionally, further studies should be conducted to confirm the efficacy and tolerability of long-term clinical application.

## Figures and Tables

**Table 1:** Characteristics of the included studies.

Included trials	Eligibility criteria	Intervention groups	Gender Female/male (*n*)	Mean age (years)	White ethnic *n* (%)	Migraines per month Mean (SD)	Efficacy outcome	Adverse events
Kuca et al., 2018 [23]	IHS 1.1 and 1.2.1 (3^rd^ edition)	Placebo	525/92	42.4	479 (77.6%)	5.1 (1.8)	Headache pain-free; MBS free;	Dizziness; fatigue
		Lasmiditan 100 mg	512/118	42.2	471 (74.8%)	5.1 (1.8)	Sustained pain-freedom at 24 h/48 h;	Paresthesia; nausea
		Lasmiditan 200 mg	515/94	41.4	450 (73.9%)	5.3 (2.3)	Headache relief at 2 h; nausea/vomiting/phonophobia/photophobia-free at 2 h	Lethargy; somnolence
Färkkilä et al., 2012 [22]	IHS 1.1 and 1.2.1 (2^nd^ edition)	Placebo	75/11	40.5	86 (100%)	3.1 (1.7)	Headache response at 2 h	Dizziness; fatigue
		Lasmiditan 50 mg	69/13	40.4	81 (99%)	3.3 (1.6)	Pain-free at 2 h	Paresthesia; nausea
		Lasmiditan 100 mg	68/14	42.0	81 (99%)	3.3 (1.7)	Headache recurrence within 24 h	Vertigo
		Lasmiditan 200 mg	65/6	39.5	70 (99%)	3.3 (1.9)	Rescue drug 2-24 h; patient's global	Sensation of heaviness
		Lasmiditan 400 mg	65/5	38.7	69 (99%)	3.1 (1.6)	Impression (much or very much better) at 2 h	Somnolence
Ferrari et al., 2010 [21]	IHS 1.1 and 1.2.1 (2^nd^ edition)	Placebo	38/4	40.3	42 (100%)	3.3	Headache response at 2 h	Dizziness; fatigue
		Lasmiditan 20 mg (iv)	24/4	38.9	28 (100%)	3.3	Pain-free at 2 h	Paresthesia
		Lasmiditan 30 mg (iv)	14/2	40.3	16 (100%)	3.5	Sustained pain response	Sensation of heaviness
		Lasmiditan 45 mg (iv)	3/1	40.8	4 (100%)	2.8	Sustained pain free; nausea/photophobia/phonophobia 2 h	Feeling of relaxation
Goadsby et al., 2019 [24]	IHS 1.1 and 1.2.1 (3^rd^ edition)	Placebo	545/100	42.6	516 (80.0%)	5.5 (2.4)	Headache pain-free at 2 h	Dizziness; fatigue
		Lasmiditan 50 mg	554/100	42.8	524 (80.1%)	5.2 (2.0)	MBS-free at 2 h	Paresthesia; nausea
		Lasmiditan 100 mg	539/96	43.4	509 (80.2%)	5.3 (1.9)	Sustained pain-free at 24 h	Somnolence
		Lasmiditan 200 mg	536/113	41.8	522 (80.4%)	5.3 (1.9)	Headache pain relief at 2 h; nausea/phonophobia/photophobia/vomiting free at 2 h	Lethargy

IHS: International Headache Society; MBS: most bothersome symptom; SD: standard deviation; iv: injection of vein.

**Table 2:** RRs and 95% CI of patient's absence of migraine-associated symptoms at 2 h after the first dose.

Comparisons	Nausea-free at 2 h			Vomiting-free at 2 h			Photophobia-free at 2 h			Phonophobia-free at 2 h		
	*N*	RR (95% CI)	*I* ^2^	*N*	RR (95% CI)	*I* ^2^	*N*	RR (95% CI)	*I* ^2^	*N*	RR (95% CI)	*I* ^2^
*Placebo comparisons*												
Lasmiditan 50 mg vs. placebo	3	0.99 (0.94, 1.05)	0%	2	1.02 (0.94, 1.11)	72%	3	1.19 (1.04, 1.37)^∗^	15%	3	1.13 (1.05, 1.22)^∗^	0%
Lasmiditan 100 mg vs. placebo	3	1.04 (1.00, 1.08)	46%	3	1.01 (0.98, 1.04)	81%	3	1.35 (1.17, 1.57)^∗^	72%	3	1.16 (1.10, 1.22)^∗^	8%
Lasmiditan 200 mg vs. placebo	3	1.03 (0.99, 1.07)	0%	3	1.00 (0.99, 1.01)	0%	3	1.30 (1.21, 1.40)^∗^	10%	3	1.16 (1.10, 1.22)^∗^	0%
*Dosage comparisons*												
Lasmiditan 50 mg vs. 100 mg	2	0.96 (0.91, 1.01)	0%	2	0.98 (0.94, 1.02)	56%	2	0.91 (0.84, 0.98)^∗^	41%	2	0.94 (0.88, 1.01)	18%
Lasmiditan 100 mg vs. 200 mg	3	1.00 (0.97, 1.05)	18%	3	1.01 (0.99, 1.03)	67%	3	1.00 (0.94, 1.05)	19%	3	1.00 (0.96, 1.05)	0%
Lasmiditan 200 mg vs. 50 mg	2	1.02 (0.96, 1.08)	0%	2	1.00 (0.98, 1.02)	0%	2	1.12 (1.04, 1.22)^∗^	0%	2	1.06 (0.99, 1.14)	0%

^∗^ *P* < 0.05 (significant difference). *N*: number of trials; RR: risk ratio; 95% CI: 95% confidence intervals.

**Table 3:** RRs and 95% CI for secondary outcomes of lasmiditan at 2 h after the first dose.

Comparisons	Headache relief at 2 h			Use of rescue medication (2–24 h)			No/mild disability at 2 h			Global impression of change (very much/much better) at 2 h		
	*N*	RR (95% CI)	*I* ^2^	*N*	RR (95% CI)	*I* ^2^	*N*	RR (95% CI)	*I* ^2^	*N*	RR (95% CI)	*I* ^2^
*Placebo comparisons*												
Lasmiditan 50 mg vs. placebo	3	1.27 (1.15, 1.41)^∗^	0%	3	0.88 (0.72, 1.07)	0%	3	1.13 (1.03, 1.25)^∗^	0%	3	1.33 (1.13, 1.55)^∗^	0%
Lasmiditan 100 mg vs. placebo	3	1.51 (1.25, 1.82)^∗^	75%	3	0.75 (0.61, 0.92)^∗^	0%	3	1.23 (1.08, 1.40)^∗^	58%	3	1.60 (1.42, 1.81)^∗^	25%
Lasmiditan 200 mg vs. placebo	3	1.41 (1.28, 1.54)^∗^	20%	3	0.81 (0.66, 0.99)^∗^	0%	3	1.16 (1.03, 1.30)^∗^	46%	3	1.62 (1.43, 1.82)^∗^	0%
*Dosage comparisons*												
Lasmiditan 50 mg vs. 100 mg	2	0.81 (0.60, 1.09)	73%	2	1.20 (0.94, 1.55)	41%	2	0.90 (0.82, 0.98)^∗^	0%	2	0.86 (0.75, 0.99)^∗^	38%
Lasmiditan 100 mg vs. 200 mg	3	1.01 (0.95, 1.08)	22%	3	0.95 (0.77, 1.18)	0%	3	1.05 (0.99, 1.13)	0%	3	0.99 (0.90, 1.09)	0%
Lasmiditan 200 mg vs. 50 mg	2	0.98 (0.72, 1.34)	67%	2	1.04 (0.82, 1.33)	36%	2	0.94 (0.86, 1.03)	0%	2	1.16 (1.01, 1.34)^∗^	0%

^∗^ *P* < 0.05 (significant difference). *N*: number of trials; RR: risk ratio; 95% CI: 95% confidence intervals.

**Table 4:** RRs and 95% CI for the most common TEAEs during the evaluation period after the first dose.

Comparisons	Dizziness			Paresthesia			Fatigue			Somnolence			Nausea		
	*N*	RR (95% CI)	*I* ^2^	*N*	RR (95% CI)	*I* ^2^	*N*	RR (95% CI)	*I* ^2^	*N*	RR (95% CI)	*I* ^2^	*N*	RR (95% CI)	*I* ^2^
*Placebo comparisons*															
Lasmiditan 50 mg vs. placebo	3	3.54 (1.22, 10.23)^∗^	64%	3	3.35 (1.59, 7.04)^∗^	45%	3	3.05 (1.58, 5.87)^∗^	0%	2	2.86 (1.60, 5.09)^∗^	0%	2	8.38 (1.55, 45.39)^∗^	0%
Lasmiditan 100 mg vs. placebo	3	5.96 (2.77, 12.79)^∗^	69%	3	3.90 (2.43, 6.26)^∗^	21%	3	6.99 (3.62, 13.48)^∗^	0%	3	2.59 (1.70, 3.95)^∗^	0%	3	4.15 (0.82, 21.00)	60%
Lasmiditan 200 mg vs. placebo	3	6.58 (3.49, 12.39)^∗^	57%	3	5.03 (3.17, 7.99)^∗^	0%	3	6.77 (3.51, 13.07)^∗^	0%	3	2.92 (1.92, 4.42)^∗^	0%	3	4.01 (1.49, 10.76)^∗^	23%
*Dosage comparisons*															
Lasmiditan 50 mg vs. 100 mg	2	0.63 (0.33, 1.18)	77%	2	0.38 (0.22, 0.65)^∗^	0%	2	0.64 (0.40, 1.01)	0%	2	1.08 (0.71, 1.64)	0%	2	0.74 (0.35, 1.54)	0%
Lasmiditan 100 mg vs. 200 mg	3	0.85 (0.67, 1.06)	40%	3	0.76 (0.57, 1.03)	0%	3	1.02 (0.74, 1.42)	0%	3	0.88 (0.65, 1.20)	0%	3	0.82 (0.36, 1.85)	60%
Lasmiditan 200 mg vs. 50 mg	2	0.50 (0.39, 0.65)^∗^	0%	2	3.15 (1.87, 5.30)^∗^	28%	2	0.58 (0.37, 0.91)^∗^	0%	2	0.83 (0.56, 1.24)	0%	2	0.78 (0.29, 2.10)	26%

^∗^ *P* < 0.05 (significant difference). *N*: number of trials; RR: risk ratio; 95% CI: 95% confidence intervals.

## Data Availability

The data used to support the findings of this study are included in the article and supplementary material files.

## Supplementary Materials

Supplementary 1 S1 File: PRISMA 2009 checklist.

Supplementary 2 S2 File: search strategies.

Supplementary 3 S3 File: forest plot: the proportion of migraineurs reporting TEAE (at least one) after the first dose between different dosage comparisons.
