# Supplementary material for: The Effect and Safety of 5-HT1F Receptor Agonist Lasmiditan on Migraine: A Systematic Review and Meta-Analysis
Source: Biomed Res Int. 2021 Oct 7;2021:6663591. doi: 10.1155/2021/6663591 (PMC8517628; doi:10.1155/2021/6663591)
Supplement: Supplementary 2 — S2 File: search strategies. [file 6663591.f2.doc]

**Search strategy for Pubmed**

#1 "Migraine Disorders"[Mesh]

#2 (((((((((((((((((((((((((((((((((Disorder, Migraine[Title/Abstract]) OR (Disorders, Migraine[Title/Abstract])) OR (Migraine Disorder[Title/Abstract])) OR (Migraine[Title/Abstract])) OR (Migraines[Title/Abstract])) OR (Migraine Headache[Title/Abstract])) OR (Headache, Migraine[Title/Abstract])) OR (Headaches, Migraine[Title/Abstract])) OR (Migraine Headaches[Title/Abstract])) OR (Acute Confusional Migraine[Title/Abstract])) OR (Acute Confusional Migraines[Title/Abstract])) OR (Migraine, Acute Confusional[Title/Abstract])) OR (Migraines, Acute Confusional[Title/Abstract])) OR (Status Migrainosus[Title/Abstract])) OR (Hemicrania Migraine[Title/Abstract])) OR (Hemicrania Migraines[Title/Abstract])) OR (Migraine, Hemicrania[Title/Abstract])) OR (Migraines, Hemicrania[Title/Abstract])) OR (Migraine Variant[Title/Abstract])) OR (Migraine Variants[Title/Abstract])) OR (Variant, Migraine[Title/Abstract])) OR (Variants, Migraine[Title/Abstract])) OR (Sick Headache[Title/Abstract])) OR (Headache, Sick[Title/Abstract])) OR (Headaches, Sick[Title/Abstract])) OR (Sick Headaches[Title/Abstract])) OR (Abdominal Migraine[Title/Abstract])) OR (Abdominal Migraines[Title/Abstract])) OR (Migraine, Abdominal[Title/Abstract])) OR (Migraines, Abdominal[Title/Abstract])) OR (Cervical Migraine Syndrome[Title/Abstract])) OR (Cervical Migraine Syndromes[Title/Abstract])) OR (Migraine Syndrome, Cervical[Title/Abstract])) OR (Migraine Syndromes, Cervical[Title/Abstract])

#3 #1 OR #2

#4 "lasmiditan" [Supplementary Concept]

#5 ((((2,4,6-trifluoro-N-(6-((1-methylpiperidin-4-yl)carbonyl)pyridin-2yl) benzamide [Title/Abstract]) OR (Reyvow[Title/Abstract])) OR (lasmiditan hydrochloride [Title/Abstract])) OR (COL-144[Title/Abstract])) OR (LY573144[Title/Abstract])

#6 #4 OR #5

#7 “randomized controlled trial[Publication Type] OR randomized[Title/Abstract] OR placebo[Title/Abstract]”

#8 #3 AND #6 AND #7
