# Supplementary figures and images for: The Effect and Safety of 5-HT1F Receptor Agonist Lasmiditan on Migraine: A Systematic Review and Meta-Analysis
Source: Biomed Res Int. 2021 Oct 7;2021:6663591. doi: 10.1155/2021/6663591 (PMC8517628; doi:10.1155/2021/6663591)

**a 50 mg versus 100 mg**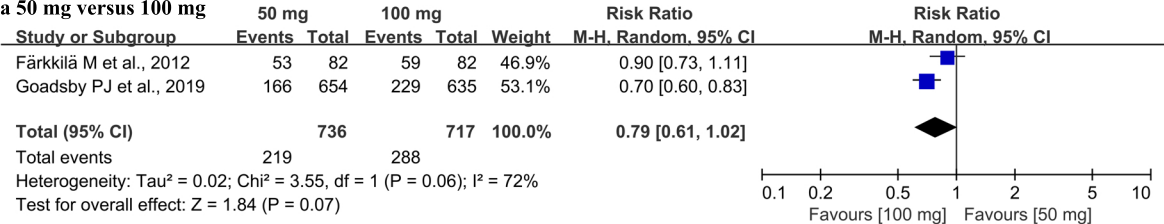**b 100 mg versus 200 mg**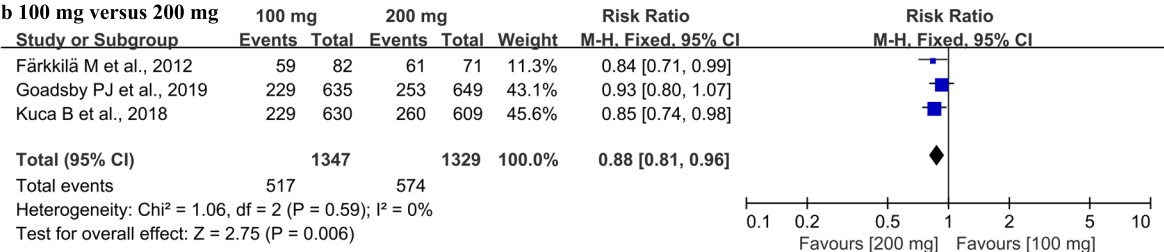**c 200 mg versus 50 mg**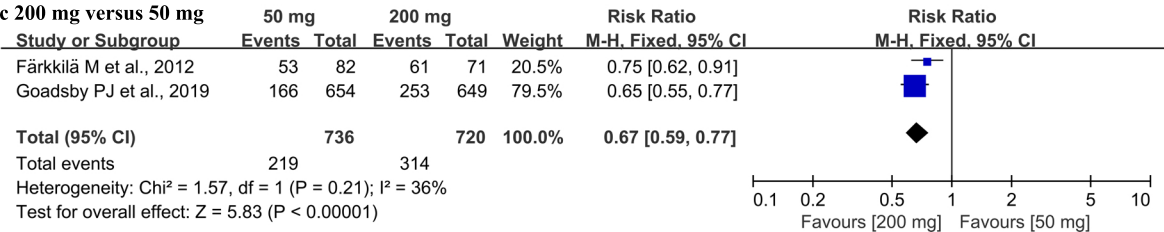

Supplement: Supplementary 3 — S3 File: forest plot: the proportion of migraineurs reporting TEAE (at least one) after the first dose between different dosage comparisons. [file 6663591.f3.pdf]
